# Whole-Genome Sequences of Salmonella enterica Serovar I 4,[5],12:i:− Isolates from Swine

**DOI:** 10.1128/MRA.00223-19

**Published:** 2019-05-23

**Authors:** Selma Gonzalez, Roger B. Harvey, H. Morgan Scott, Sara D. Lawhon, Javier Vinasco, Leonardo Mariño-Ramírez, Keri N. Norman

**Affiliations:** a Veterinary Integrative Biosciences, Texas A&M University, College Station, Texas, USA; b Food and Feed Safety Research Unit, Agricultural Research Service, U.S. Department of Agriculture, College Station, Texas, USA; c Veterinary Pathobiology, Texas A&M University, College Station, Texas, USA; d National Center for Biotechnology Information, National Library of Medicine, National Institutes of Health, Bethesda, Maryland, USA; University of Arizona

## Abstract

Salmonella enterica (non-Typhi) is one of the top five pathogens causing enteric infections worldwide. Draft whole-genome sequences of multidrug-resistant (MDR) Salmonella enterica serovar I 4,[5],12:i:− isolates from swine tissue samples collected at slaughter were evaluated for antimicrobial resistance genotypes. In recent years, Salmonella enterica serovar I 4,[5],12:i:− has increased in prevalence in humans and animals and has been linked to the consumption of contaminated pork.

## Abstract (precis)

Salmonella enterica (non-Typhi) is one of the top five pathogens causing enteric infections worldwide. Draft whole-genome sequences of multidrug-resistant (MDR) Salmonella enterica serovar I 4,[5],12:i:− isolates from swine tissue samples collected at slaughter were evaluated for antimicrobial resistance genotypes.

## ANNOUNCEMENT

Salmonella enterica (non-Typhi) is one of the most common foodborne pathogens and is the causative agent of salmonellosis in humans. There are nearly 1.2 million illnesses and 450 deaths related to Salmonella infections every year in the United States (1, 2). Common gastrointestinal symptoms that develop with salmonellosis in humans are diarrhea, abdominal pain, and fever (3). However, serious infections can lead to hospitalization or death, particularly in young children, adults over 65 years old, and those who are immunocompromised.

There are a limited number of *Salmonella* serovars that are responsible for the majority of cases of human salmonellosis (4). In particular, multidrug-resistant (MDR) Salmonella enterica serovar I 4,[5],12:i:− has been on the rise in both humans and animals, as reported by the National Antimicrobial Resistance Monitoring System (NARMS) (5). An increasing prevalence of antimicrobial resistance has been observed in nontyphoidal *Salmonella* over recent decades (3, 6). The recent rise in MDR nontyphoidal *Salmonella* infections is largely driven by the increase in Salmonella enterica serovar I 4,[5],12:i:− with resistance to ampicillin, streptomycin, sulfonamide, and tetracycline (ASSuT) (7). The isolates described here are Salmonella enterica serovar I 4,[5],12:i:− isolates cultured by the United States Department of Agriculture Agricultural Research Services (USDA ARS, College Station, TX) from swine tissue samples collected at slaughter.

Salmonella enterica serovar I 4,[5],12:i:− isolates were cultured using standard culture and enrichment techniques from swine head trim and cheek meat samples (8). Samples were collected every other month from January to November 2015 from a pork processing plant in the United States and shipped to the USDA ARS (College Station, TX). Previously cultured isolates were streaked onto tryptic soy agar with 5% sheep blood and incubated at 37°C for 18 hours (Remel, Lenexa, KS). A single colony was selected and inoculated into 5 ml of tryptic soy broth for DNA extraction. DNA isolation was performed using QIAmp 96 DNA QIAcube HT kits and the QIAcube HT instrument (Qiagen, Valencia, CA) (9). DNA quality and quantity were verified by DNA absorbance and fluorescence using the FLUOstar Omega plate reader (BMG Labtech, Cary, NC). Whole-genome sequencing libraries were prepared using Nextera XT library preparation kits (Illumina, San Diego, CA) with a fragment length of 600 bp and sequenced on the Illumina MiSeq platform using MiSeq reagent kits v3 with 2 × 300-bp paired-end reads. Default parameters were used for all bioinformatics software unless otherwise specified. FastQC v0.11.07 was used to assess the quality of the raw Illumina reads and determine the total number of reads generated (10). Trimmomatic v0.36 was used to remove adapter/primer sequences, low-quality bases, and reads with a quality threshold of 3 for reading and trailing, using the following parameters: 4:15 SLIDINGWINDOW, minimum length of 36 bases, and a Phred quality score of 33 (11). The genomes were assembled using the *de novo* assembler SPAdes v3.5 (12), and the quality of assembly was evaluated using QUAST (v4.6) (13). Resistance genes were found using the raw sequencing data in ResFinder (v3.0) (14). The National Center for Biotechnology Information (NCBI) Prokaryotic Genome Annotation Pipeline (PGAP) was used to annotate the genomes (15). Phenotypic antimicrobial susceptibility patterns of Salmonella enterica serovar I 4,[5],12:i:− isolates were identified by broth microdilution on a Sensititre system (Trek, Thermo Scientific Microbiology, Oakwood Village, OH), using Gram-negative National Antimicrobial Resistance Monitoring System (NARMS) CMV3AGNF plates, and defined as susceptible, intermediate, or resistant, according to Clinical and Laboratory Standards Institute (CLSI) MIC breakpoints or to NARMS breakpoints when CLSI criteria are not established.

A total of 45 out of 47 Salmonella enterica serovar I 4,[5],12:i:− isolates sequenced were found to be phenotypically MDR, defined as resistant to 3 or more antibiotic classes. Forty isolates were found to harbor *bla*_TEM-1_, *strA*, *strB*, *sul2*, and *tet*(B), commonly known as the ampicillin, streptomycin, sulfonamide, and tetracycline resistance type (R-type ASSuT). We found that 32 isolates with the R-type ASSuT also contained the plasmid-mediated quinolone resistance (PMQR) gene *qnrB*. Three isolates harbored additional resistance genes to streptomycin-spectinomycin (*aadA2*), aminoglycosides [*aph(3′)-I*], sulfonamides (*sul1*), beta-lactams (*bla*_SHV-12_), and tetracyclines [*tet*(D)]. Future comparative analyses will advance our understanding of genome evolution and multidrug resistance in Salmonella enterica serovar I 4,[5],12:i:−.

### Data availability.

This whole-genome shotgun sequencing project has been deposited in DDBJ/EMBL/GenBank under the accession numbers shown in Table 1. The raw sequencing reads have been submitted to the SRA database under the accession number SRP139298, and the BioProject accession number is PRJNA449363. The versions described in this paper are the first versions.

**TABLE 1:** Sequencing information for isolates described in this study

Isolate name	SRA accession no.	BioSample no.	GenBank accession no.	No. of reads	No. of contigs	Total sequence length (bp)	GC content (%)	*N*_50_ (bp)
A65HEB1	SRR6977162	SAMN08897954	QBFS00000000	989,228	100	5,045,691	52.7	195,649
B19CEB2.1	SRR6977164	SAMN08897956	QBFU00000000	945,158	114	4,956,390	52.3	170,808
B51HEB1.2	SRR6977163	SAMN08897957	QBFV00000000	1,627,028	75	4,942,762	52.3	282,875
B68HEB2.1	SRR6977158	SAMN08897958	QBFW00000000	1,904,792	81	4,944,241	52.9	272,046
B70HEB1.1	SRR6977157	SAMN08897959	QBFX00000000	1,137,224	136	5,045,346	52	144,984
B87HEB1.1	SRR6977160	SAMN08897960	QBFY00000000	1,140,012	88	4,956,943	52.1	232,430
B9HEB1.1	SRR6977161	SAMN08897955	QBFT00000000	1,846,422	83	4,971,860	52.6	282,782
C12C1	SRR6977156	SAMN08897962	QBGA00000000	2,021,810	87	4,973,021	51.9	232,430
C55H1	SRR6977155	SAMN08897963	QBGB00000000	2,512,246	110	4,985,991	52.2	272,046
C6C1	SRR6977159	SAMN08897961	QBFZ00000000	2,106,342	96	4,975,489	52.2	278,603
E51C1	SRR6977154	SAMN08897964	QBGC00000000	1,978,954	131	4,983,350	52.3	223,309
E53H1	SRR6977153	SAMN08897965	QBGD00000000	1,627,904	122	4,979,610	52.1	232,430
E55H1	SRR6977152	SAMN08897966	QBGE00000000	884,928	127	4,980,599	52.7	171,138
E58H1	SRR6977151	SAMN08897967	QBGF00000000	1,828,272	108	4,980,841	52.3	257,316
E61C1	SRR6977150	SAMN08897968	QBGG00000000	1,943,844	131	4,984,203	52.1	265,813
E62H2	SRR6977149	SAMN08897969	QBGH00000000	1,083,666	111	4,981,960	52.7	174,666
E64C1	SRR6977148	SAMN08897970	QBGI00000000	1,087,462	131	4,982,693	52.8	174,714
E65H1	SRR6977147	SAMN08897971	QBGJ00000000	1,039,444	128	4,981,903	52.8	150,132
E67H1	SRR6977146	SAMN08897972	QBGK00000000	726,000	146	4,981,604	52.6	114,427
E68C1	SRR6977145	SAMN08897973	QBGL00000000	1,473,952	109	4,980,055	51.8	223,309
E68C2	SRR6977132	SAMN08897974	QBGM00000000	1,341,830	121	4,981,708	51.9	265,813
E68C3	SRR6977133	SAMN08897975	QBGN00000000	1,218,018	144	4,983,832	52.2	181,598
E69C1	SRR6977130	SAMN08897976	QBGO00000000	1,497,898	125	4,981,993	52	265,813
E72C1	SRR6977131	SAMN08897977	QBGP00000000	1,687,610	124	4,981,747	51.7	265,813
E72H1	SRR6977128	SAMN08897978	QBGQ00000000	1,390,846	120	4,982,357	51.9	265,813
E74C1	SRR6977129	SAMN08897979	QBGR00000000	696,300	172	4,978,670	52.9	84,564
E83C1	SRR6977126	SAMN08897980	QBGS00000000	867,328	173	4,977,795	53	78,499
E87C1	SRR6977127	SAMN08897981	QBGT00000000	1,223,600	89	4,914,455	52.4	185,367
E93C1	SRR6977134	SAMN08897982	QBGU00000000	1,019,664	160	4,982,906	53	99,642
E97C1	SRR6977135	SAMN08897983	QBGV00000000	661,886	199	4,977,804	53	69,192
F62C1	SRR6977144	SAMN08897984	QBGW00000000	1,930,414	92	4,956,596	52.2	232,430
F63H1	SRR6977143	SAMN08897985	QBGX00000000	1,536,274	95	4,958,940	52.1	270,591
F63H2	SRR6977124	SAMN08897986	QBGY00000000	1,132,436	81	4,956,430	51.9	270,591
F63H3	SRR6977119	SAMN08897987	QBGZ00000000	767,568	156	4,956,156	53.1	87,734
F64H1	SRR6977140	SAMN08897988	QBHA00000000	1,545,292	95	4,961,867	52	267,386
F64H2	SRR6977139	SAMN08897989	QBHB00000000	2,114,612	86	4,960,400	52.2	270,591
F64H3	SRR6977142	SAMN08897990	QBHC00000000	1,970,406	143	4,963,436	53	91,060
F65H1	SRR6977141	SAMN08897991	QBHD00000000	930,644	90	4,960,505	52	270,591
F65H2	SRR6977137	SAMN08897992	QBHE00000000	784,614	128	4,959,713	52.9	101,887
F65H3	SRR6977136	SAMN08897993	QBHF00000000	1,137,968	149	4,970,338	52.1	222,314
F70H1	SRR6977122	SAMN08897994	QBHG00000000	1,602,208	123	5,239,330	51.8	272,046
F70H2	SRR6977123	SAMN08897995	QBHH00000000	578,698	161	5,234,191	52.6	90,408
F70H3	SRR6977165	SAMN08897996	QBHI00000000	1,168,474	117	5,237,241	51.7	251,287
F72H1	SRR6977125	SAMN08897997	QBHJ00000000	2,190,780	89	4,961,754	52.1	270,591
F72H2	SRR6977138	SAMN08897998	QBHK00000000	1,232,914	104	4,960,932	52.8	171,532
F72H3	SRR6977120	SAMN08897999	QBHL00000000	603,506	153	4,955,922	52.9	84,597
F73C1	SRR6977121	SAMN08898000	QBHM00000000	1,181,774	108	4,961,534	52.9	164,165
